# In Memoriam—Julius G. Schmitt, M. D.

**Published:** 1894-05

**Authors:** 


### IN MEMORIAM—JULIUS G. SCHMITT, M. D.

SYRACUSE, N. Y., March 15th, 1894.

Resolutions of the Central New York Homœopathic Medical Society upon the death of the late Julius G. Schmitt.

WHEREAS, We learn with surprise and great sorrow, of the death of Dr. Julius G. Schmitt, an honored, active, and influential member of this Society, who died March 2d, 1894; therefore,

*Resolved*, That we extend our heartfelt sympathy to his wife, and to his friends in Germany, and to his friends in Rochester and elsewhere, believing his death to be a cause for sincere grief;

*Resolved*, That these Resolutions be entered on the minutes of this Association, and that copies be furnished Mrs. Schmitt, *The Advance*, and THE HOMŒOPATHIC PHYSICIAN.

T. D. STOW,
A. B. CARR,
*Committee.*

